# Immunogenicity of a newly developed vaccine against *Clostridium perfringens* alpha-toxin in rabbits and cattle

**DOI:** 10.14202/vetworld.2022.1617-1623

**Published:** 2022-07-07

**Authors:** Mohamed J. Saadh, Feras F. Lafi, Adnan A. Dahadha, Mohamed S. Albannan

**Affiliations:** 1 Department of Pharmacy, Faculty of Pharmacy, Middle East University, Amman, Jordan; 2 Department of Genetic Engineering and Biotechnology, Faculty of Science, Philadelphia University, Jordan; 3 Department of Research and development, Biotechnology Research Center, 23 July St., Industrial Zone, New Damietta, 34517, Egypt

**Keywords:** alpha-toxin, *Clostridium perfringens* A, potency, safety, stability, toxoid

## Abstract

**Background and Aim:**

*Clostridium perfringens* type A is an anaerobic bacterium that produces four major toxins (alpha, beta, epsilon, and iota) that cause various diseases. Most of the important *C. perfringens*-associated diseases of farm animals are caused by alpha-toxin. This study aimed to produce a vaccine against alpha-toxin using *C. perfringens* type A (ATCC 13124) and investigate its potency, stability, and safety.

**Materials and Methods:**

The vaccine was formulated of its constituents for 1 h. Each milliliter of the final vaccine product contained alpha toxoid 15 lecithovitellinase activity (Lv) by adding (0.375 mL containing 40 Lv) and approximately 0.2 mL from 3% concentrated aluminum hydroxide gel, <0.001% W/V thiomersal, <0.05% W/V formaldehyde, and nearly 0.425 mL phosphate-buffered saline (pH 7.2). The vaccine efficacy was evaluated in rabbits and cattle by performing potency, stability, and safety tests.

**Results:**

The vaccine produced approximately 8.8 and 4.9 IU/mL neutralizing antibodies in rabbits and cattle, respectively. These concentrations were higher than the lowest concentration recommended by various international protocols and the United States Department of Agriculture by 2.20-fold in rabbits and 1.23-fold in cattle. Interestingly, the formulated vaccine enhanced immune responses by 1.80-fold in rabbits compared with that in cattle; the difference was statistically significant (p < 0.0001). The vaccine was stable for 30 months. In vaccinated rabbits, the body temperature slightly increased temporarily during the first 10 h of vaccination; however, the temperature difference was not statistically significant (p > 0.05).

**Conclusion:**

This study describes a manufacturing process to obtain sufficient amounts of a vaccine against *C. perfringens* alpha-toxin. The formulated vaccine effectively elicited a higher level of neutralizing antibody response than the international standards. Furthermore, the vaccine was found to be stable, safe, and effective in preventing *C. perfringens*-related diseases in rabbits and cattle. Further studies are necessary to evaluate the efficacy of this vaccine in other farm animals.

## Introduction

*Clostridium perfringens* is an anaerobic, spore-forming bacterium pathogenic to both animals and humans [1, 2]. This Gram-positive ubiquitous species is primarily found in ground and surface water or soil [3] and secretes a mixture of enzymes and toxins responsible for myonecrosis [4]. *C. perfringens* is one of the most pathogenic species in the *Clostridium* genus and can produce at least 20 toxins [3]. Overall, *C. perfringens* strains are categorized into seven toxinotypes (A–G) based on the genotypic expression of four major toxins (alpha, beta, epsilon, and iota). In mammals, including humans, most diseases associated with *C. perfringens* are caused by the aforementioned four types of toxins. However, two minor toxins – enterotoxin or necrotic enteritis B-like toxin – secreted by certain *C. perfringens* strains lead to necrotic enteritis in broiler chickens or food poisoning in humans [5]. The type A strains are mainly commensal and widespread in the environment. However, all *C. perfringens* strains can produce alpha-toxin [6]. Alpha-toxin is a zinc-dependent phospholipase C (molecular weight: 43 kDa) that has two domains and can degrade eukaryotic cell membranes [7]. More specifically, this toxin hydrolyzes the phosphodiester bond between the polar head and non-polar tail of the phospholipid membrane, which facilitates pathogenesis [8]. In both animals and humans, alpha-toxin is the main causative agent of gas gangrene, in which removal of the infected and damaged tissues becomes necessary. In general, curing infections induced by *C. perfringens* toxins are highly improbable; thus, vaccination against the toxins is the standard process of infection control. Vaccination is the most competent medical process that can substantially reduce the incidence of infections that may otherwise result in millions of deaths worldwide [9]. Although the development of vaccines using conventional methods is well known and systematic, the actual production can be challenging because of the high-cost and long-term experiments necessary to verify the benefits and safety of these vaccines in humans. Therefore, there is an urgent need for an alternative, novel, and economically feasible process for vaccine development.

In light of the above–mentioned background, the present study aimed to develop an effective method to produce sufficient amounts of a vaccine against *C. perfringens* alpha-toxin and evaluate the efficacy (potency, stability, and safety) of this formulated vaccine in eliciting immune response in rabbits and cattle.

## Materials and Methods

### Ethical approval

All animal experiments were performed in accordance with the guidelines of the National Council for Animal Experimentation Control, and the Ethical Committee approval was obtained from Ethical Committee of Middle East University-Jordan.

### Study period and location

This study was conducted from March 2019 to August 2020 in Jordan Bio-Industries Center (JOVAC), Amman, Jordan.

### Sample

A strain of *C. perfringens* type A (ATCC 13124) was obtained from JOVAC, Amman, Jordan, for vaccine formulation.

### Preparation of culture medium

The culture medium for fermentation was prepared by mixing 3 g yeast extract, 1 g starch, 10 g meat extract, 5 g D-glucose, 3 g sodium acetate, 10 g bacteriological peptone, 0.1 g sodium bisulfate, and 5 g sodium chloride with 1 L of distilled water. The medium was autoclaved for 15 min at 121°C under 1.1 bar and then cooled at 25°C. The pH was adjusted to 6.6–7.0.

### Growth conditions of *C. perfringens* type A used to produce toxins for veterinary vaccine development

The contents of a freeze-dried working seed vial (1 × 10^9^ colony-forming units [CFUs]) were suspended in 2 mL of the aforementioned diluted medium. The culture was anaerobically incubated at 37°C for 12 h. Subsequently, a subculture was produced using 75 mL of this culture in a blue-colored cap bottle; this served as the inoculum for 20 L of the culture medium in the fermenter at 37°C for 11 h. Throughout fermentation, oxygen-free nitrogen was insufflated to maintain an anaerobic state and 40% sodium hydroxide was added with regular agitation at 105 rpm to maintain the pH at 7.2–7.4.

### Inactivation

Maximum bacterial growth was noted after 22 h of incubation at 37°C. *C. perfringens* type A was inhibited by adding formaldehyde to ensure a final concentration of 0.2% toxin per 1 L of culture in the fermenter with continuous stirring at 33°C for 2 days. If necessary, 20% of sodium bisulfite was added to neutralize the remaining formaldehyde after inactivation. The inactivated culture of *C. perfringens* type A was centrifuged at 28,620× *g*; the supernatant was collected.

### Vaccine formulation

Before inactivation, the culture was sampled to assess lecithovitellinase activity (Lv). From this supernatant, 375 mL (40 Lv/mL) was taken and diluted using 425 mL phosphate-buffered saline (PBS) (pH = 7.2). Thiomersal was added (0.001% W/V) to obtain the final concentration. In addition, 200 mL 3% aluminum hydroxide [Al (OH)_3_] gel (equivalent to 5.18 mg of aluminum) was added (20% V/V) and agitated continuously for approximately 1 h to obtain a final concentration of 15 Lv/mL. Furthermore, the pH was adjusted to 6.8–7.2.

### Titration test for C. perfringens type A

Next, 0.5 mL of *C. perfringens* type A culture was added to 4.5 mL PBS (pH = 7.2) to achieve a dilution of 10^−1^, which was further diluted serially to 10^−2^, 10^−3^, 10^−4^, 10^−5^, 10^−6^, and 10^−7^. Inoculation was performed using 0.1 mL of 10^−6^ and 10^−7^ serially diluted tubes in three plates of Reinforced Clostridial Agar independently, which were incubated at 37°C for 3 or 4 days. Then, the number of growing colonies in each plate was counted, and the mean value was calculated by multiplying with the dilution factor to obtain the desired last titer in CFU/mL.

### Inactivation kinetics of the culture medium

The extent of inactivation was assessed every 4 h on day 1 and every 8 h on following days to determine the total inactivation time. Formaldehyde was used as the inactivation factor for vaccine production. Next, 20 mL of each sample was centrifuged at 28,650× *g* for approximately 15 min. Subsequently, the supernatant was removed and the pellet resuspended in sterile peptone water. The last step was repeated thrice to eliminate the residual formaldehyde and perform the titration test.

### Inactivation kinetics of alpha-toxin (toxigenesis test)

In total, 100 mL sample was retrieved every 12, 24, 36, 48, 72, and 96 h while maintaining the culture temperature at 37°C. Each sample was centrifuged at 28,620× g for 15 min, and the supernatant containing the toxin was consecutively filtrated using 0.45 and 0.2 mm filters. The remaining formaldehyde was removed from the supernatant by introducing the samples to various consecutive concentrations and dilutions with normal saline solution. Toxigenesis was assessed by calculating the lethal dose (LD_50_/mL) for each sample. The samples were diluted with normal saline at the following rations: 1:100, 1:200, 1:400, 1:800, and 1:1600. Next, five mice (body weight: 18–22 g) were intravenously injected with 0.25 mL of each dilution according to the European Pharmacopoeia guidelines [10]. Mice were then observed for a period of 1 week, and the toxin titer was calculated (LD_50_/mL) using the Spearman-Karber method [11].

### Determining the presence of lecithovitellinase

This test was performed using *C. perfringens* type A culture before inactivation to determine Lv. For this, 1 mL of sample toxins was incubated at room temperature (RT) (25°C) for 40 min with 1 mL of diluted alpha antitoxin standard for *C. perfringens* (National Institute for Biological Standards and Control [NIBSC]). A fixed volume of lecithovitellinase was added to each tube, mixed, and incubated at 37°C for 60 min. The tubes were then left on the laboratory bench overnight at 25°C, and their opalescence was evaluated visually. The mixture showed approximately 30–40% opalescence and contained alpha-toxin units equivalent to the units of antiserum used. If the result was negative, this process was repeated using higher or lower units [12, 13].

### Potency test

A potency test was performed using a total of 20 female New Zealand rabbits aged 3–6 months with an approximate body weight of 1.5 kg. These rabbits were categorized into two groups, each comprising 10 rabbits. The first group was injected with the formulated vaccine, whereas the negative control group was injected with a mixture of sterile PBS with 20% Al(OH)_3_ gel. Furthermore, five cows were injected with the vaccine; the negative control group for cows was injected with a mixture of sterile PBS and Al (OH)_3_. On days 0 and 21, 10 rabbits and 5 cows received 1 and 3 mL of the formulated vaccine, respectively. Blood was drawn on day 35. Blood collection was performed for all groups using a sterile tube, and the blood samples were allowed to clot at 25°C (RT) to obtain serum through centrifugation. Serum samples were collected from all groups and pooled [14–17], and seroneutralization assays were performed. Seroneutralization assays were also performed using the serum samples obtained from rabbits and cattle after 3 months. The potency test was repeated every 3 months up to 12 months and every 6 months up to 30 months to study vaccine stability. A total of 80 rabbits and 40 cows were used for this test; 10 rabbits and five cows were comprised as the respective control groups.

The processes followed for estimating the alpha antitoxin levels were in accordance with the United States Department of Agriculture (USDA), European Pharmacopoeia, and Code of Federal Regulations Title 9 (CFR-9) guidelines.

### Seroneutralization assay

To measure beta antitoxin levels (IU/mL), 1 mL of each standard toxin (NIBSC) was incubated at 37°C for 1 h with 1 mL of diluted pooled sera (dilution range: 1:1–1:32). Next, 10 mice weighing 18–22 g were intravenously injected with 0.2 mL of each sample and were subsequently observed for 72 h for survival; the mice were euthanized if necessary. The procedure was repeated with intermediary dilutions of the sera to identify the lowest dilution exerting protective effects on the infected mice. The procedures performed were based on the USDA [18], British Pharmacopoeia, European Pharmacopoeia [19–21], and CFR-9 [19] guidelines for measuring beta antitoxins.

### Safety test

Safety tests were performed using 20 rabbits (young rabbits, 15; adult rabbits, 5). All of these rabbits were free of specific antibodies against the antigens targeted by the formulated vaccine. All adult and young rabbits were administered with the final vaccine, and their rectal temperature was measured to determine whether the vaccine was safe and if any side effects occurred in the immunized animals. In general, the rabbits were categorized into four groups based on the administrated therapeutic dose: Group 1 (five young rabbits that received 1 mL vaccine), Group 2 (five young rabbits that received a double dose), Group 3 (five young rabbits that were immunized twice; the duration between the two vaccinations was 21 days); and Group 4 (five adult rabbits that received one dose) [22].

### Sterility test

Sterility tests were performed using eight tubes containing 0.5 mL of the formulated vaccine, four tubes containing 20 mL of thioglycolate broth, and four tubes containing 20 mL of Sabouraud Dextrose Broth. Two tubes containing thioglycolate broth were anaerobically added to the incubation medium. The contents of the remaining tubes were aerobically added to the incubation medium. All tubes were incubated at 37°C for 3 weeks for sterility assessment.

### Statistical analysis

Statistical analysis was performed using the Statistical Package for the Social Sciences software version 15.0(IBM Corp., NY, USA) and GraphPad Prism package version 5.0 (www. https://www.graphpad.com/support/prism-5-updates/). Continuous variables were described as mean ± standard deviation (SD) values. Analysis of variance and Student’s t-test were performed to estimate between-group statistical differences. p < 0.05 was considered statistically significant and p < 0.0001 was considered highly significant.

## Results

**Figure-1:**
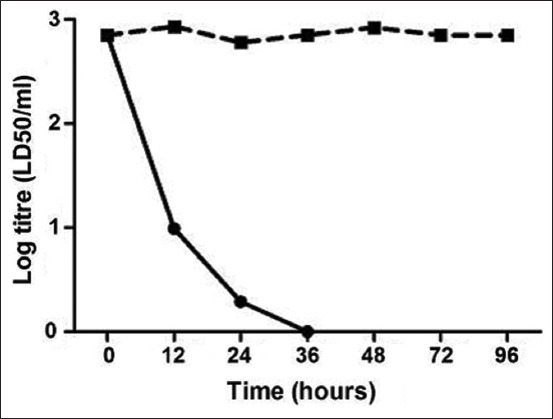
The inactivation kinetic. Its respective alpha-toxin compared with those without any inhibition. Formaldehyde was employed as a neutralizing factor in manufacturing vaccines. The inactivation operation of alpha-toxin was tested every 12 h through the first 48 h and later each 24 h.

**Figure-2:**
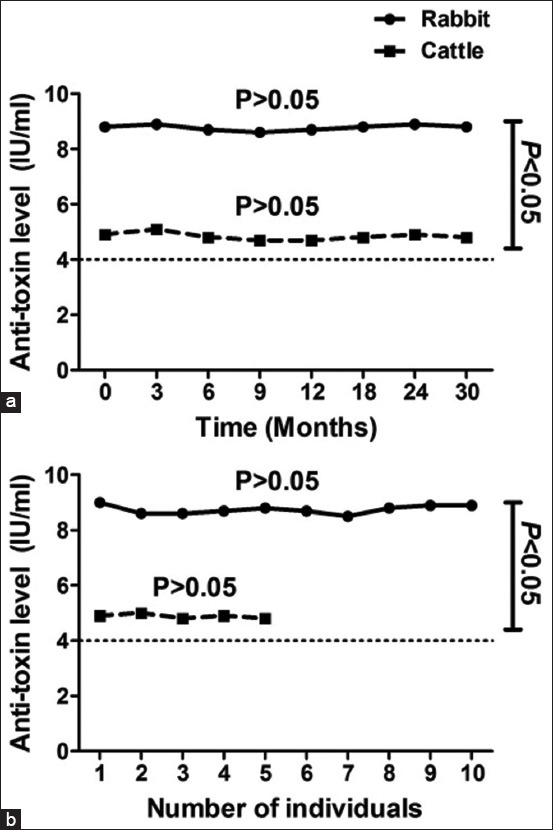
The distribution of antitoxin concentrations discovered based on the potency test in both rabbits and cattle after vaccination (a) on employing pooled serum and (b) on employing each serum individually. The dotted line indicates the minimum antitoxin level (4 IU/mL) suggested by the United States Department of Agriculture. Our results appeared that the formulated vaccine was able to stimulate higher immune responses in rabbits than in cattle with an extremely considerable variation (p < 0.0001).

**Figure-3:**
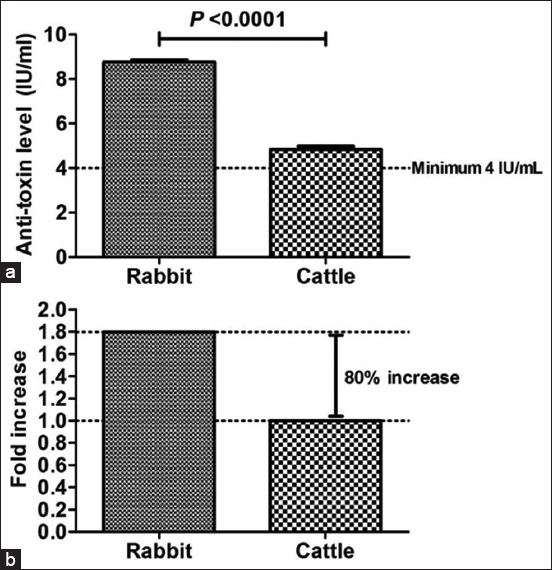
Scales of anti-toxins antibodies till vaccination in various animal models (a) mean value of antitoxin levels detected in rabbits and cattle after immunization at various intervals during 30 months and (b) distribution of observed fold changes for anti-toxins antibodies in rabbits and cattle. The minimum antitoxin level suggested by the United States Department of Agriculture was 4 IU/mL.

*C. perfringens* type A bacteria were inactivated using 5 mL of 35–40% formaldehyde with continuous agitation at 33°C, and 20% sodium bisulfite was added to remove the residual formaldehyde. The extent of inactivation was then assessed every 4 h on day 1 and every 8 h on the following days to determine the total inactivation time. Surprisingly, *C. perfringens* type A bacteria were totally inhibited after 48 h. The aforementioned operations were obligatory to neutralize the possible side effects by detoxification of the toxin and reducing bacterial replication. By calculating the median lethal dose (LD_50_/mL) every 12 h using various samples (100 mL each), the toxigenic profile was determined (Figure-1). Various dilutions (1:100, 1:200, 1:400, 1:800, and 1:1600) of the toxin were subcutaneously injected into five mice (body weight: 18–22 g) to determine the total inactivation time. The toxin was noted to have completely converted into its toxoid (inactivated) form after 36 h (Figure-1). The inhibited alpha toxoid was emulsified (1:1) in 3% Al(OH)_3_ suspension (pH = 6.8–7.2; adjuvant) and incubated at 25°C overnight with continuous stirring. Stability tests were performed throughout the vaccine development process and after 3, 6, 9, 12, 18, 24, and 30 months by observing the physical manifestations of the formulated vaccine in animals as well as the pH of the samples. The vaccine maintained a yellowish-brown color with a mean (± SD) pH of 6.96 (± 0.03; minimum pH: 6.8 and maximum pH: 7.1). To check for contamination of the formulated vaccine, sterility tests were performed; results indicating negative growth in both aerobic and anaerobic conditions suggest non-contamination. Subsequently, a potency test was performed using female New Zealand rabbits (age: 3–6 months; body weight: approximately 1.5 kg). Serum samples obtained from each group were tested. The titer of neutralizing antibodies in mice was determined through seroneutralization; the results of the potency test are shown in Figure-2a. To confirm the results obtained using rabbits as the experimental model, another animal species (cattle) was used for vaccination. Figure-2b shows the response of the animal models to immunization. The formulated vaccine surpassed the minimum antitoxin level (4 IU/mL) defined by the USDA as the production titer of the neutralizing antibody against alpha-toxin was 8.8 (± 0.16) IU/mL in rabbits and 4.9 (± 0.08) IU/mL in cattle. The target antibody concentration in rabbits was increased by 120% (i.e., 2.20-fold) over the minimum antitoxin concentrations. The antibody concentration was lower in cattle than in rabbits but increased by 23% (i.e., 1.23-fold) over the minimum antitoxin concentrations. Interestingly, the vaccine elicited 1.80-fold higher immune responses in rabbits than in cattle (i.e., 80% increase); the difference was significant (p < 0.0001; Figures-3a and b).

To evaluate the safety profile of the vaccine, three groups of adult and young rabbits were immunized (Table-1). No immunized rabbits exhibited any local or systemic adverse reactions. A slight increase in the mean rectal temperature was observed in the vaccinated rabbits during the first 10 h of vaccination, but the difference was not significant (p > 0.05). The temperature was reduced to the normal level after 24 h (Table-1).

**Table 1:** The rectal temperature measurement after every vaccination in various groups of rabbits.

Group	Treatment	Time (h)							p-value^c^

		24 h before	0 h	10 h	24 h	48 h	72 h	96 h	
Group I^a^	One dose	38.8 ± 0.6	38.7 ± 0.6	39.2 ± 0.6	38.6 ± 0.5	38.9 ± 0.6	38.7 ± 0.6	38.7 ± 0.6	p > 0.05
Group II^a^	Double dose	38.5 ± 0.7	38.5 ± 0.6	39.1 ± 0.7	38.8 ± 0.7	39.1 ± 0.6	38.6 ± 0.4	38.5 ± 0.4	p > 0.05
Group III^a^	Repeated dose^b^	38.6 ± 0.5	38.6 ± 0.6	39.0 ± 0.7	38.5 ± 0.5	39.0 ± 0.6	38.6 ± 0.8	38.7 ± 0.5	p > 0.05
Group IV^a^	One dose	38.7 ± 0.5	38.6 ± 0.6	39.0 ± 0.5	38.6 ± 0.5	38.8 ± 0.5	38.5 ± 0.6	38.4 ± 0.7	p > 0.05
p-value^c^		p > 0.05	p > 0.05	p > 0.05	p > 0.05	p > 0.05	p > 0.05	p > 0.05	

Continuous variables were expressed as mean ± SD.

a Groups I, II, and III: Young rabbits, Group IV: Adult rabbits.

b Grou III was administered an extra dose of vaccine after 21 days.

c p > 0.05 is considered non-significant; p< 0.05 is considered statistically significant

## Discussion

The present study demonstrated an effective method for producing sufficient amounts of a vaccine against *C. perfringens* alpha-toxin. The results indicate that this method ensures the elicitation of immune responses as well as enhancement of immune defenses in various animal models. Alpha-toxins of *C. perfringens* are essential virulence agents that ensure bacterial survival in both animal and human hosts. Intoxication due to a clostridial protein may lead to cell death; despite the severity of infection, there are hardly any therapeutic agents that are effective against this toxin [6]. Thus, immunization is the most preferred method for managing this disease. The actual application of any formulated vaccine depends on several factors: The vaccine must be nonpathogenic in experimental animal models and able to produce high titers of antibodies; furthermore, it must provide immunity following inoculation and show thermal stability without any local and systemic adverse reactions. The present study used an easier-to-implement approach to formulate a vaccine against the alpha-toxin of *C*. *perfringens* type A (ATCC 13124). This vaccine was safe and produced high levels of alpha-toxin. This method may eliminate major challenges faced during the manufacturing of these vaccines. Notably, this vaccine did not induce adverse reactions in the experimental animal models. Furthermore, this cost-effective vaccine produced a high number of neutralized antibodies, elicited defensive immunity after inoculation, and facilitated thermal stability without any local or systemic side effects.

In recent years, *C. perfringen*s strains have been identified to produce various forms of toxins such as alpha-toxin, beta2-toxin, and perfringolysin O. The virulence agents may not always be toxins; these may be proteins produced based on specific characteristics of *C. perfringen*s causing gastric infection. The vaccines produced against *C. perfringens* type A may provide immunity against all types of related proteins and toxins. Unfortunately, recombinant vaccines can provide immunity only against alpha-toxin [5, 10]. Although recombinant vaccines against *C. perfringens* alpha-toxin may not be inactivated after purification, stability studies are lacking. Furthermore, the recombinant alpha-toxin used in the present study was stored after lyophilization protein. This lyophilized protein was suspended in PBS and then incubated with AL(OH)_3_ under weak mixing conditions for 20 h at 25°C for homogenization. Satisfactory absorbance of the proteins on the surface of Al(OH)_3_ correlates with infection risk and enhanced intricacy of the immunization process and does not lead to efficient outcomes [21].

Initially, *C. perfringens* type A, as well as its respective alpha-toxin, was inactivated by the addition of formaldehyde. This is because formaldehyde – a one-carbon, highly hydrophilic aldehyde – can eliminate the damaging effects, reduce bacterial replications, and detoxify bacterial toxins [22, 23]. A question then emerges regarding the exact of the formulated vaccine that can elicit and maintain immune responses. The answer to the aforementioned question may be obtained based on the elevated titer of the neutralized antibodies, which surpassed the minimum desired antitoxin level of 4.8 IU/mL on inoculation in rabbits. Interestingly, the newly formulated vaccine exhibited a 2.20-fold increase over the minimum antitoxin level. The above-mentioned results suggest that this novel production method effectively elicits neutralized antibody response higher than the international standards (120% increase).

Another question is as follows: “What is the effect of this vaccine on various animal models? Can the aforementioned results be reproduced using another animal model?” Thus, this study focused on estimating the range within which the formulated vaccine elicited an immune response in cattle. The vaccine’s efficiency was lower in cattle than in rabbits, but the titer of the neutralized antibodies in cattle was higher than the international standards. Interestingly, the formulated vaccine exhibited a 1.23-fold increase over the minimum antitoxin level in cattle. Of note, this vaccine elicited an immune response more efficiently in rabbits than in cattle; the difference was significant. That might be because the titers of the neutralized antibodies produced in rabbits – the animal model used for evaluating the vaccine against *C. perfringens* alpha-toxin – were evidently higher than those noted in cattle. The vaccine exhibited immune responses 1.80-fold higher in rabbits than in cattle (80% increase); the difference was significant (p < 0.0001). In line with the findings of this study, evidence reports a recombinant vaccine that produces 9.6 and 5.19 IU/mL neutralizing antibodies in rabbits and cattle, respectively [17, 24]. Consistent with the findings of the present study, a similar previous study conducted in 2016 indicated a 1.80-fold decrease in immune response (antibody titer) in cattle than in rabbits [21, 23]. In the present study, the difference between rabbits and cattle was 3.90 IU/mL, which coincides with the value reported by a previous study on alpha-toxin [21]. The present study is the first based on a series of studies assessing the stability and safety profiles of the alpha-toxin vaccine. The vaccine maintained its stability for a period of 30 months without changes in potency in both rabbits and cattle. Furthermore, none of the tested animals exhibited any symptoms of toxicity or allergic reactions against the formulated vaccine. The formulated vaccine caused a slight increase in the body temperature of the experimental animals. However, the temperature returned to the normal level after 24 h.

## Conclusion

This study described an effective method for producing sufficient amounts of a vaccine against *C. perfringens* alpha-toxin. The vaccine was effective in eliciting a higher level of neutralizing antibody response than the international standards. In addition, the vaccine was stable for a prolonged period after production (up to 2 years). Although further studies are necessary to demonstrate its efficacy, this method represents an effective and safe approach for the production of vaccines to prevent *C. perfringens*-related diseases in rabbits and cattle.

## Authors’ Contributions

MJS: Designed the study and drafted the manuscript. MJS, AAD and FFL: Performed all the experimental procedures. MJS, AAD and FFL: Formulated the vaccine. MJS and MSA: Conducted data analysis and interpretation. All authors have read and approved the final manuscript.
